# The mRNA cap methyltransferase gene *TbCMT1* is not essential *in vitro* but is a virulence factor *in vivo* for bloodstream form *Trypanosoma brucei*

**DOI:** 10.1371/journal.pone.0201263

**Published:** 2018-07-24

**Authors:** Anna Kelner, Michele Tinti, Maria Lucia S. Guther, Bernardo J. Foth, Lia Chappell, Matthew Berriman, Victoria Haigh Cowling, Michael A. J. Ferguson

**Affiliations:** 1 Wellcome Centre for Anti-Infectives Research, School of Life Sciences, University of Dundee, Dundee, United Kingdom; 2 The Wellcome Trust Sanger Institute, Hinxton, United Kingdom; 3 Centre for Gene Regulation and Expression, School of Life Sciences, University of Dundee, Dundee, United Kingdom; University of Cambridge, UNITED KINGDOM

## Abstract

Messenger RNA is modified by the addition of a 5′ methylated cap structure, which protects the transcript and recruits protein complexes that mediate RNA processing and/or the initiation of translation. Two genes encoding mRNA cap methyltransferases have been identified in *T*. *brucei*: *TbCMT1* and *TbCGM1*. Here we analysed the impact of *TbCMT1* gene deletion on bloodstream form *T*. *brucei* cells. *TbCMT1* was dispensable for parasite proliferation in *in vitro* culture. However, significantly decreased parasitemia was observed in mice inoculated with *TbCMT1* null and conditional null cell lines. Using RNA-Seq, we observed that several cysteine peptidase mRNAs were downregulated in *TbCMT1* null cells lines. The cysteine peptidase Cathepsin-L was also shown to be reduced at the protein level in *TbCMT1* null cell lines. Our data suggest that *TbCMT1* is not essential to bloodstream form *T*. *brucei* growth *in vitro* or *in vivo* but that it contributes significantly to parasite virulence *in vivo*.

## Introduction

*Trypanosoma brucei*, a protozoan parasite transmitted by the tsetse fly, causes Human African Trypanosomiasis (HAT) and Nagana in cattle [1]. When a tsetse fly feeds on infected human or animal blood, stumpy form *T*. *brucei* trypomastigotes enter the insect midgut and differentiate into the proliferating procyclic form (PCF) cells [2]. These migrate to the salivary glands and differentiate into proliferating epimastigote form cells and finally into non-dividing animal-infective metacyclic trypomastigotes [3]. During a subsequent blood meal, the tsetse fly transmits the metacyclic trypomastigotes into the hemolymphatic system of the host where they transform into the rapidly proliferating slender bloodstream form (BSF), and the cycle of infection begins again.

*T*. *brucei* genes are arranged in polycistronic units. RNA Polymerase II (RNA Pol II) transcribes protein-coding genes into polycistrons containing dozens of transcripts [4, 5]. These polycistrons are unidirectional and partitioned by strand-switch regions (SSRs) that are characterised by a stretch of G nucleotides between divergent stretches of genes. The processing of the transcription unit occurs co-transcriptionally by trans-splicing coupled to cleavage of the 3’ end by the polyadenylation machinery for poly(A) addition [6]. During trans-splicing, a capped 39-nucleotide (nt) spliced leader (SL), or mini-exon, is added to the 5′ termini of mRNAs [7]. This SL is independently transcribed from a tandem array of 140-nt SL RNA genes [8]. In addition to acting as a splicing substrate for the excision of a mRNA from the primary transcript, the SL RNA also provides each protein-coding mRNA with a 5′ cap structure. In eukaryotic cells, the mRNA cap includes *N*-7-methylguanosine (m7G) linked to the first transcribed nucleotide via a 5' to 5' triphosphate linkage [9]. Trypanosomatids exhibit additional unique processing of the first four transcribed nucleotides in a structure called cap4 [10]. In cap4, the first four nucleotides are modified by 2′-*O*-ribose methylations as well as additional base methylations on the first and fourth nucleotides, to create the structure m^7^Gpppm^6^_2_AmpAmpCmpm^3^Um [8, 11].

The cap4 structure of SL RNA is required for *trans*-splicing and is therefore essential for the production of mature mRNAs. The enzymes which catalyse cap formation in eukaryotes consist of a triphosphatase and guanylyltransferase, which catalyse guanosine cap addition, and a series of methyltransferases which methylate the guanosine cap and transcribed nucleotides. In *T*. *brucei*, the guanylyltransferase (*TbCE1*), which transfers GMP to the diphosphate end of RNA, had no noticeable effect on PCF *T*. *brucei* cell viability when downregulated by RNAi [12, 13]. Two genes encoding *N*-7 guanosine cap methyltransferases have been identified in *T*. *brucei*: *TbCMT1* and *TbCGM1*. *TbCMT1* is a monofunctional cap methyltransferase. *TbCGM1* has cap methyltransferase activity and guanylyltransferase activity, which presumably compensates when *TbCE1* is suppressed [13–15]. Quantitative proteomics demonstrated that the expression of *TbCMT1* is equivalent in PCF and BSF life stages of *T*. *brucei* [16], and RNAi-mediated suppression of *TbCMT1* also did not result in a growth defect in PCF cells [13]. Here we investigated the function of *TbCMT1* in BSF cells.

## Results

### *TbCMT1* is not required for Lister 427 BSF proliferation *in vitro*

To investigate the role of *TbCMT1* in BSF cells, null and conditional-null mutants of *TbCMT1* were generated in a Lister 427 BSF ‘single marker’ (SM) cell line. The SM cell line has been genetically modified to express the tetracycline repressor protein (TetR) and T7 RNA polymerase, both under G418 selection [17]. In this paper, we will refer to the SM cell line as wild type (WT) cells. To make the tetracycline-inducible *TbCMT1* conditional-null clones, the first *TbCMT1* allele was replaced with *PAC* to create a *TbCMT1* heterozygote and an ectopic copy of the *TbCMT1* gene, fused to a C-terminal MYC_3_-tag, was subsequently introduced into the rDNA locus under phleomycin selection using the pLew100 vector. Following induction with tetracycline, the second *TbCMT1* allele was replaced with *HYG* (Panel A in S1 Fig). The *TbCMT1* null clones were created by replacing the remaining *TbCMT1* allele of the *TbCMT1* heterozygote with an *HYG* gene by homologous recombination (S2 Fig). Samples of genomic DNA of the mutant cell lines and their intermediates were analysed by Southern blot to confirm the loss of the endogenous *TbCMT1* alleles and the correct genomic locations of the *PAC* and *HYG* genes (Panel B in S1 Fig and S2 Fig).

**Fig 1:**
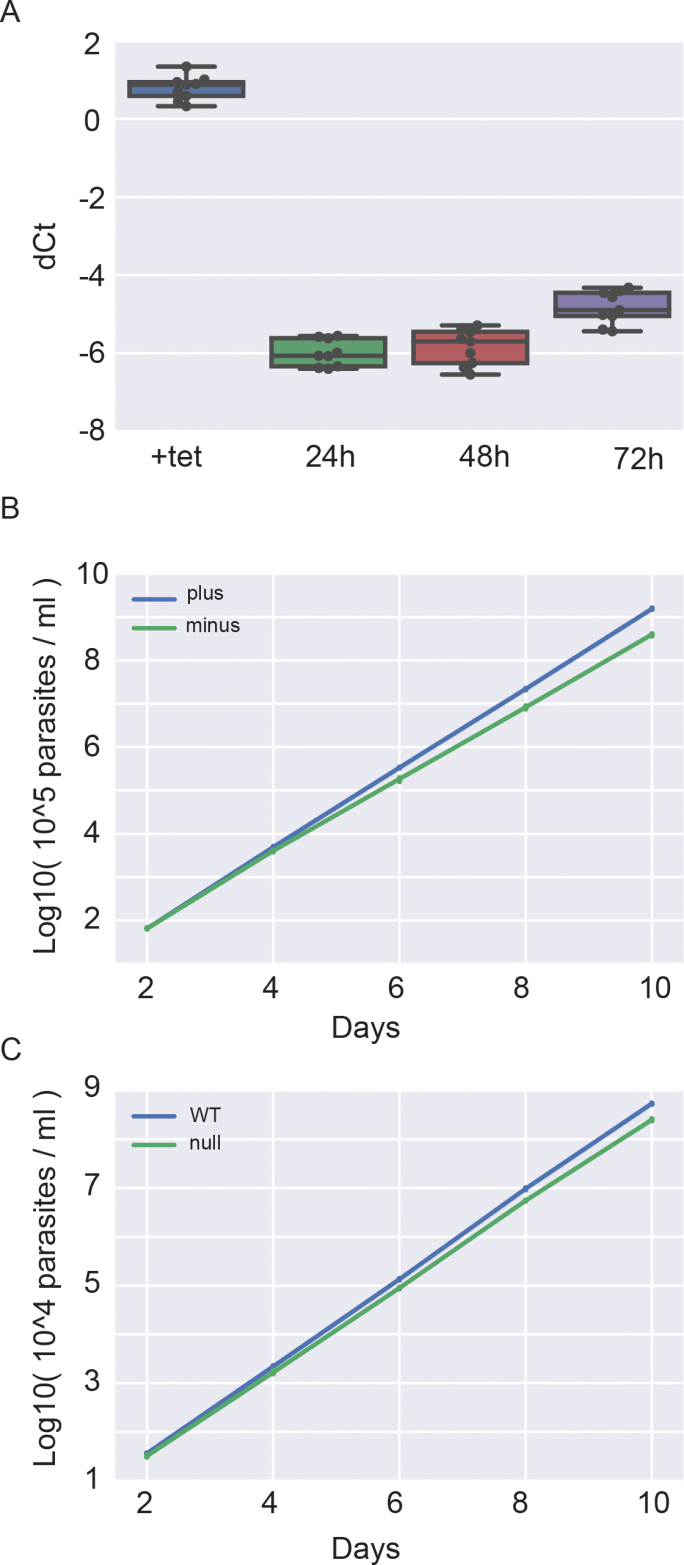
*TbCMT1* is not required for *T*. *brucei* Lister 427 BSF proliferation in cell culture. (A) Total RNA was purified from *TbCMT1* conditional null cells cultured with (+tet) and without tetracycline for 24, 48 and 72 h. *TbCMT1* expression was analysed by qRT-PCR normalised to telomerase reverse transcriptase (*TERT*). The delta Ct values (dCt) for nine measurements for each condition are visualised as a swarm plot to show all observations along with representations of the underlying distributions. (B) Cumulative cell counts of triplicate *TbCMT1* conditional null mutant cell cultures grown with (plus) and without (minus) tetracycline. (C) Cumulative cell counts of triplicate wild type (WT) and *TbCMT1* null cells cultured in parallel. For the data in panels B and C, the cell counts of three biological replicates are reported after 2, 4, 6, 8 and 10 days. The cultures were counted and diluted to 10^5^ cells/ml every two days in (B) and to 10^4^ cells/ml every two days in (C). The cell counts are reported as the log10 value of the cumulative number of parasites per ml of cell culture allowing for the aforementioned dilution factors.

The tetracycline-dependence of *TbCMT1-MYC*_*3*_ mRNA expression in the conditional null cell line was analysed. After 24 h without tetracycline, less than 1% *TbCMT1* transcript was detected by qRT-PCR compared to the cells grown in the presence of tetracycline (Fig 1A). The rate of BSF cell proliferation was measured under permissive (plus tetracycline) and non-permissive (minus tetracycline) conditions over ten days. Lack of *TbCMT1-MYC*_*3*_ transcription slightly impaired cell proliferation, indicating that *TbCMT1* is likely to be dispensable for *T*. *brucei* survival *in vitro* (Fig 1B). However, since the fetal bovine serum used to culture the WT cell line might contain traces of tetracycline or doxycycline, we decided to make *TbCMT1* null mutants and determined their growth characteristics. As observed for the *TbCMT1* conditional null cell line, deletion of the *TbCMT1* gene only slightly impaired cell proliferation in culture (Fig 1C), confirming unambiguously that *TbCMT1* is not essential for *T*. *brucei* survival *in vitro*.

### The absence of *TbCMT1* expression reduces *T*. *brucei* Lister 427 BSF proliferation *in vivo*

**Fig 2:**
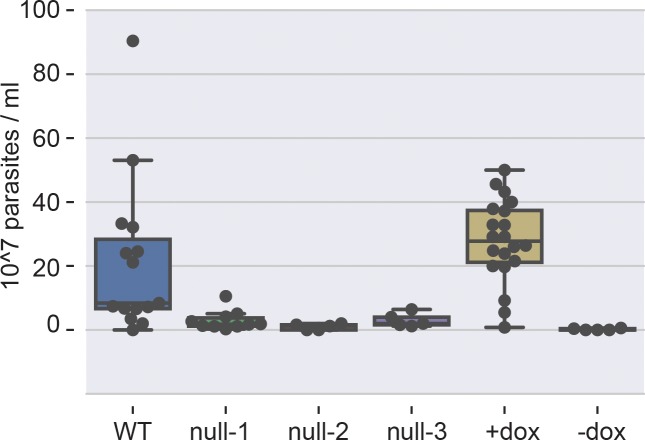
*TbCMT1* is required for *T*. *brucei* proliferation *in vivo*. Mice were inoculated with wild type (WT) parasites, three independent clones of *TbCMT1* null mutants (null-1, null-2 and null-3), and a *TbCMT1* conditional-null clone (c-null). Mice receiving the latter were dosed with (+dox) or without (-dox) doxycycline in the drinking water for seven days before and following inoculation. Blood parasitemias were measured in triplicate for each animal three days after infection and a total of 14 (WT), 5 (null-1), 5 (null-2), 10 (null-3), 20 (c-null +dox) and 5 (c-null -dox) animals were used. The parasitemia is reported as the number of parasites per ml of blood.

Deletion of some *T*. *brucei* genes can affect parasite growth *in vivo* but not *in vitro* [18, 19] and *vice versa* [20, 21]. To determine the role of *TbCMT1 in vivo*, we investigated whether deletion of this gene influences *T*. *brucei* parasitemia in mice. Mice were inoculated with WT cells, three independent clones of *TbCMT1* null cells, and a conditional null clone under-permissive (*i*.*e*., with doxycycline in the drinking water) and non-permissive conditions. The conditional null cells were fully activated with tetracycline in culture prior to their injection into mice. Three days after infection, WT *T*. *brucei* had proliferated as expected while all three *TbCMT1* null clones exhibited a significant reduction in proliferation (Fig 2). The *TbCMT1* conditional null cells were similarly defective in proliferation in the absence, but not in the presence, of doxycycline (Fig 2). Taken together, these data show that the absence of *TbCMT1* expression significantly reduces the rate of growth of bloodstream form *T*. *brucei in vivo*.

### RNA-seq analysis

**Fig 3:**
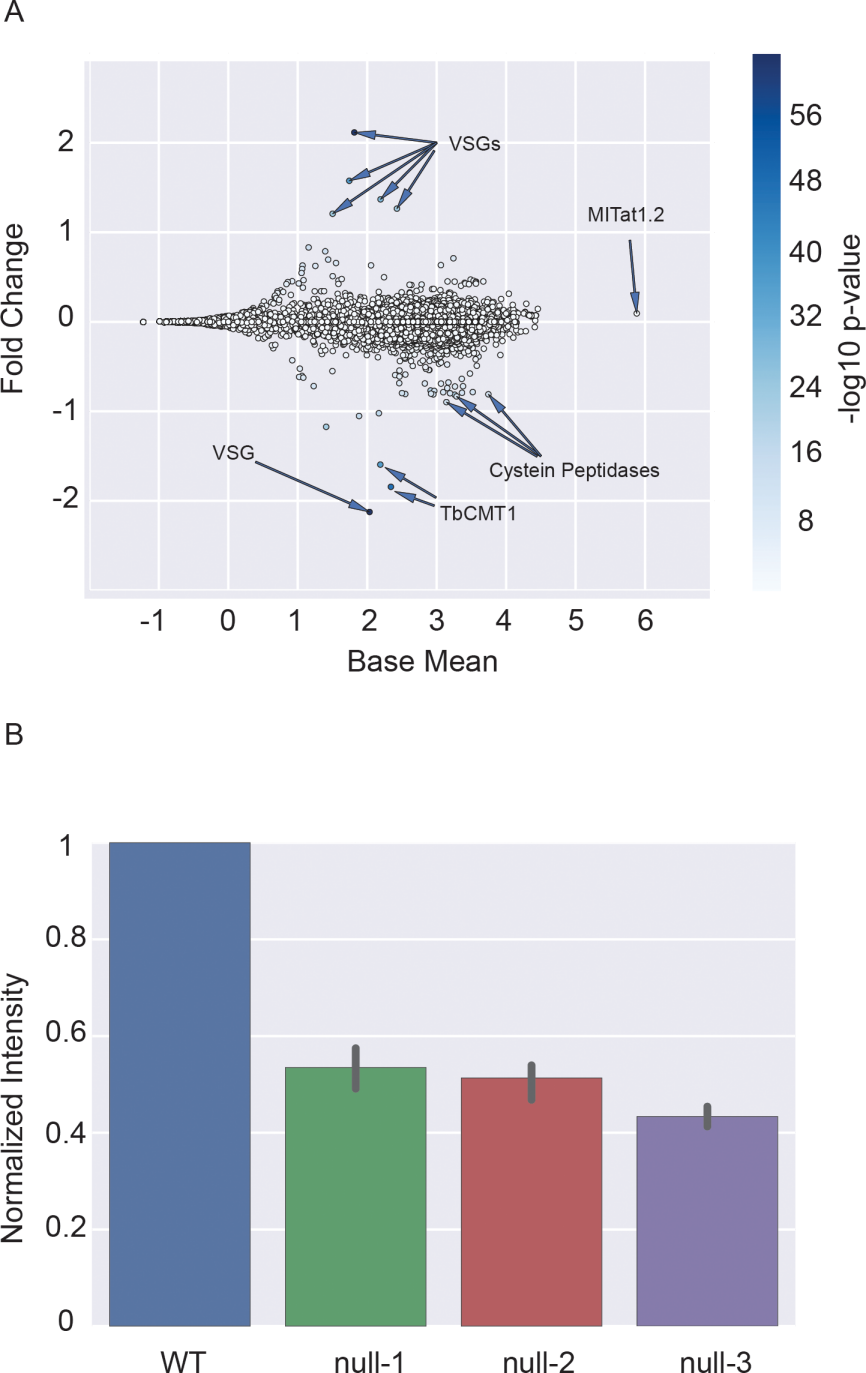
Differential gene expression analysis in wild type and *TbCMT1* null mutant cells. The figure shows the log10 transformation of the normalized mean for the transcript reads detected in the WT and *TbCMT1* null mutant samples computed by DESeq2 (Base Mean, x axis) versus the log2 transformation of their mean fold change (y axis). A positive fold change indicates transcript upregulation and a negative fold change indicates downregulation in the *TbCMT1* null samples relative to WT. Highlighted with arrows are the top 5 up-regulated VSG-related transcripts (VSGs) and top 3 down-regulated cysteine peptidases transcripts (Cysteine Peptidases). The plot also highlights the position of the top downregulated VSG transcript (VSG), the position of the downregulated *TbCMT1* transcript (TbCMT1) and the position of the unchanged MITat1.2 VSG transcript. (B) The plot shows the quantification of the Cathepsin-L protein determined by quantitative Western blot in wild type (WT) and three *TbCMT1* null mutants clones (null-1, null-2 and null-3). The values of the Cathepsin-L protein are normalized to the HSP-70 protein and divided by the value for the WT sample.

The mRNA cap and mRNA cap methyltransferases have been demonstrated to influence many processes in gene expression including transcription, RNA stability, RNA processing and initiation of translation. In human cells, a reduction in *N*-7 cap methylation has been demonstrated to have a gene-specific impact on transcript and protein levels [22, 23]. For this reason, we investigated the impact of *TbCMT1* deletion on the transcriptome. RNA was extracted from four independent WT and *TbCMT1* null mutant cell cultures and analysed by Illumina RNA sequencing. The WT and *TbCMT1* null biological replicates showed pairwise Pearson correlation coefficients >0.9 (S3 Fig). Principal component analysis (PCA) was used to evaluate the variance between each biological replicate (S4 Fig) and this showed good separation of the WT and *TbCMT1* null biological replicates in the second principal component. For this reason, all of the samples were used for the differential expression analysis, which revealed 86 differentially regulated genes with p-values <0.01 (S1 Table). Although at first inspection it appeared that *TbCMT1* (Tb927.10.4500) was only the second most downregulated transcript upon *TbCMT1* deletion, closer analysis of the RNA-seq coverage plot reveals that the sequence reads assigned to the gene were from the 5’ and 3’ UTRs and not from the *TbCMT1* open reading frame, confirming deletion of both *TbCMT1* alleles by homologous recombination via the 5’ and 3’ UTRs. Apart from *TbCMT1* itself, the most downregulated (and several upregulated) genes upon *TbCMT1* deletion belong to the variant surface glycoprotein (VSG) family (S1 Table and Fig 3A). The most abundant and unchanged transcript is VSG MITat1.2 which is the principal VSG expressed by the WT cell line used in these studies. However, antigenic variation (i.e., the switching of VSG gene expression) occurs stochastically and it is therefore perhaps not surprising to see low abundance VSG transcripts going up and down between samples. Other than these, the main change was the downregulation of eleven transcripts of the cysteine peptidase family. Since changes in transcript level are not always reflected in changes at the protein level [24, 25], we investigated the protein level of the cysteine peptidase cathepsin-L by Western blot in WT cells and three *TbCMT1* null clones. Cathepsin-L protein concentrations were consistently reduced by about 50% in the *TbCMT1* null clones compared to the WT cells (Fig 3B). A representative image of the Western blot analyses of Cathepsin-L using HSP-70 as an internal control is provided in (S5 Fig).

## Discussion

*TbCMT1* null *T*. *brucei* Lister 427 BSF cells proliferated similarly to the parental cell line in tissue culture. These findings are similar to the observation that RNAi-mediated knockdown of *TbCMT1* in *T*. *brucei* PCF cells also does not significantly impact cell growth [13]. This shows that *TbCMT1* is non-essential in culture. In contrast, deletion of *TbCMT1* significantly impacted parasitemia following mouse infection. Importantly, the proliferation defect of the *TbCMT1* conditional null cell line was rescued by the addition of doxycycline to the drinking water of the infected mice, providing the crucial ‘add-back’ experiment. These data implicate *TbCMT1* as a virulence gene for *T*. *brucei in vivo*, but not *in vitro*. Other examples of *T*. *brucei* genes that exert their effects more profoundly *in vivo* than *in vitro* include the two oligosaccharyltransferase genes, *TbSTT3A* and *TbSTT3B*, which are dispensable in tissue culture but are essential in mouse infections [18] and these examples underline the need to determine gene essentiality or virulence characteristics *in vivo* as well as *in vitro*.

With the aim of understanding the cellular functions affected by *TbCMT1* deletion, we performed RNA-Seq analyses of the transcriptomes of WT and *TbCMT1* null mutants. The most striking change was an approximately 2-fold downregulation of cysteine peptidase transcripts in the *TbCMT1* null mutants. The eleven *T*. *brucei* cysteine peptidase gene sequences are nearly identical, such that the sequence mapping software distributes the transcript reads arbitrarily to this group of genes. However, this reduction in transcript levels was mirrored at the protein level as judged by quantitative Western blotting using anti-Cathepsin-L antibodies. The mechanism by which *TbCMT1* deletion leads to the selective downregulation of cysteine peptidase expression is unknown. Furthermore, for technical reasons, these data were collected from the *TbCMT1* mutant grown *in vitro* and we cannot exclude the possibility that *TbCMT1* deletion has different effects *in vivo*.

Kinetoplastid parasites are known to have an abundance of cysteine peptidases that are important for their survival in their hosts [26, 27]. Further, studies in *Leishmania mexicana* have shown that cysteine peptidase-deficient amastigotes are infective *in vitro* but are significantly impaired in infectivity *in vivo* [28, 29]. Thus, the observed selective downregulation in cysteine peptidase transcript and protein levels in the *TbCMT1* mutants may explain their loss of virulence *in vivo*, although we cannot exclude additional effects of removing *TbCMT1* protein form the cells.

## Material and methods

### Cell culture

*T*. *brucei* BSF cells (strain 427, VSG variant MITaT 1.2) cells which expresses both T7 RNA polymerase and the Tetracycline repressor protein (TetR) under G418 selection [17] can be obtained through BEI Resources, NIAID, NIH: *Trypanosoma brucei* subsp. *brucei*, Strain Lister 427 VSG 221 (TetR T7RNAP) (bloodstream form), NR-42011. These are referred to as ‘wild type’ trypanosomes in this paper and were cultured at 37°C with 5% CO_2_ in the presence of at 2.5 μg/ml G418 in cell culture flasks with filter lids (Greiner). Cells were grown to a maximum density of 3x10^6^ cells/ml in HMI-9T medium. HMI-9T medium has the same formulation as HMI-9 medium (Hirumi and Hirumi, 1994), with the exceptions that 56 μM 1-thioglycerol (Sigma) is used instead of 200 μM 2-mercaptoethanol, and 2 mM GlutaMAX I (Gibco) was used instead of L-glutamine. When required the following antibiotics were used for selection, 0.1 μg/ml Puromycin, 2.5 μg/ml Phleomycin and 4 μg/ml Hygromycin. For conditional null mutant cultures, 0.5 μg /ml tetracycline was used for permissive conditions. Growth curves were obtained by counting cells from cultures using a CASY Cell Counter and Analyser Model TT (Innovatis, Roche).

### Generation of gene replacement and ectopic copy constructs

The gene sequence for *TbCMT1* (Tb927.10.4500) and its flanking regions was obtained from TriTrypDB [30]. For *TbCMT1* gene replacement by homologous recombination, about 500 bp of 5’- and 3’-UTR sequence were amplified by PCR from *T*. *brucei* genomic DNA. The sequences of the primers to amplify the 5’ UTR were: ATA AGT ATG CGG CCG CGC ACT CGC AGC GCT ATC CAG TTA TCC and *GTT TAA ACT TAC GGA CCG TCA AGC TT*T AAG GTT ACG CTT TCA CCC CTT. The sequences of the primers to amplify the 3’ UTR were: GAC GGT CCG TAA GTT TAA ACG GAT CCG GAG TAC TTA TCT CCC CGT TTT C and *ATA AGT AAG CGG CCG CG*C TGG CAT ACA GGT GAC TGG CTT C. The two PCR products were used together in a further PCR to yield a product containing the 5’-UTR linked to the 3’-UTR by a HindIII, PmeI, BamHI cloning site (italic sequences) with NotI sites at each end (underlined sequences). This was cloned into pGEM-5Zf and drug resistance genes (*HYG* and *PAC*) were cloned into the HindII and BamHI sites as described previously [25]. The constructs were verified by sequencing and digested with NotI, inactivated at 65°C for 30 min, ethanol precipitated overnight in -20°C, washed in cold 70% ethanol and resuspended in sterile water, ready for electroporation. SM BSF cells were grown to mid-log phase density at about 2x10^6^ cells/ml, harvested and resuspended at 10^7^ cells in 0.1 ml Amaxa nucleofector II containing 1μg of digested and sterile construct DNA. Sometimes, Cytomix solution (2 mM EGTA p.H. 7.6, 120 mM KCl, 0.15 mM CaCl_2_, 10 mM K_2_HPO_4_/KH_2_PO_4_ pH 7.6, 25 mM HEPES pH 7.6, 5 mM MgCl_2_, 0.5% Glucose, 100 μg/ml defatted BSA, 1 mM Hypoxanthine) was utilized instead of Amaxa Nucleofactor for some electroporations. Control electroporations without DNA were performed in parallel to verify antibiotic selection. Cells were electroporated using Amaxa electroporator program X-001 [31] and subsequently recovered in 12 ml HMI-9T without antibiotics for 14 h at 37°C 5% CO_2_ before addition of 12 ml of medium containing two-fold concentration of selection antibiotic (Puromycin or Hygromycin). These cultures were plated in 12 well plates (2 ml per well) for selection and incubated at 37°C plus 5% CO_2_. Antibiotic-resistant wells were cloned by dilution by plating into 96-well plates at a concentration of 1 single cell/ml to ensure the resistant cells were clonal population. Five clones of each electroporation were analysed by Southern blot [32]. To prepare the conditional null mutant, before the second *TbCMT1* allele was replaced, a tetracycline-inducible ectopic copy of the Tb*CMT1* gene in the pLEW100 vector was introduced and induced. The TbCMT1 open reading frame (ORF) was amplified with the primers: Forward ATA AGT ATC ATA TGG AGA GCC TAC GGA CTG CAG C, Reverse ATA AGT AAC TCG AGC TGC TGG CTT TCC GGA AGC ACA AC. The gene was amplified by PCR and cloned into pLEW 100-3xMYC [17]. The construct was verified by sequencing, electroporated, and recovered as described above.

### Southern blot analysis

Genomic DNA (gDNA, 5μg) was digested with restriction enzymes as indicated in S1 Fig and S2 Fig, resolved by agarose gel electrophoresis and transferred onto positively charged Nylon membrane using standard protocols. DNA probes were prepared using the PCR DIG Probe Synthesis Kit (Roche). DIG probes were checked by agarose gel against non-Dig probes of same sequence; if they showed increased size, they were presumed to be DIG labelled and used in Southern hybridisation. Hybridisation and detection (by ECL) were performed according to the manufacturer’s protocols.

### qRT-PCR

RNA was extracted from log-phase cells using RNeasy Mini Extraction Kit (Qiagen). cDNA was synthesised using the iScript cDNA Synthesis Kit (BioRad). qRT-PCR was performed using Sybrgreen master mix (Bio-Rad) in a Bio-Rad iCycler Thermal Cycler.

### Growth curve analysis

Cell cultures were inoculated at 10^4^ parasites/ml. The cultures were then counted using a CASY cell counter and diluted to 10^4^ cells/ml every two days for a total of 10 days. The cumulative cell counts of three biological replicates for each condition are reported. For the conditional null mutant, the cells were washed three times in HMI-9T minus tetracycline, diluted in the same medium to 5x10^4^ cells/ml and then cultured as described above plus and minus 0.5 μg/ml tetracycline.

### Mouse infection studies

BALB/c female adult mice were obtained from Envigo International Ltd., Huntingdon, U.K., and were housed at 21^°^C, 55–65% relative humidity with a 12 h / 12 h light / dark cycle. Groups of mice were dosed for seven days with 0.2 mg/ml doxycycline in 5% sucrose or control 5% sucrose in their drinking water prior to infection with *T*. *brucei*. Fresh drinking water solutions were provided every 48 h. Prior to infection, wild-type, *TbCMT1* null and *TbCMT1* conditional null mutants of bloodstream form trypanosomes were grown in HMI-9T media with and without tetracycline then washed in media without antibiotics and resuspended at 1x10^6^ cells/ml. Groups of 5 mice were used for each condition and each was injected intraperitoneally with 0.2 ml of cell suspension. Infections were assessed daily by tail bleeding and cell counting was performed with a Neubauer chamber and a phase-contrast microscope.

### RNA-seq analysis

Total RNA was isolated from *T*. *brucei* BSF followed by poly-A mRNA enrichment with streptavidin poly-T oligo-attached magnetic beads (Dynabeads, Invitrogen). The mRNA was then fragmented into 200 nt fragments using Covaris Adaptive Focused Acoustics process. Operating conditions—sample volume: 130 μl, duty cycle: 10%, intensity: 5, cycles per burst: 200, processing time: 60 s, water bath temperature: 4°C, power mode: frequency sweeping, degassing mode: continuous. Fragmented mRNA was concentrated by ethanol precipitation and measured on an RNA Pico chip (Agilent 2100 bioanalyzer). The first strand of cDNA was synthesised using reverse transcriptase and random primers, followed by second strand cDNA synthesis which removes the RNA template producing double-stranded cDNA. To blunt-end the DNA fragments, an end repair reaction was performed with Klenow polymerase, T4 DNA polymerase, and T4 polynucleotide kinase. A single 3’ adenosine overhang was added to the cDNA allowing the ligation of Illumina adaptors. These adaptors contain primer sites both for sequencing and complimentary annealing onto the Illumina flowcell surface. Adaptor ligated cDNA fragments were measured on an Agilent DNA chip. The final cDNA library was sequenced on a MiSeq Personal Sequencer (Illumina). The fastq files were trimmed for adaptors and assessed for the quality score distribution with the FastQC program. Reads with an average quality score less than 30 were removed from the analysis with a custom python script. In order to align reads, a hybrid genome assembly consisting of the T. brucei 927 reference genome (version 32.0) deposited at the TryTripDB database [30] and a list of 2563 distinct genes encoding complete and partial VSG genes of the Lister 427 strain [33] was assembled. The reads were aligned with bowtie2 v2.1.0 using the parameters—local—very-sensitive [34]. The Samtools package [35] was used to convert the sam files to bam files, and the Picard package was used to order the bam file by coordinate. The read count per gene was computed with Rsubread with the options isPairedEnd = TRUE and requireBothEndsMapped = TRUE [36]. The differential gene expression analysis was carried out with DESeq2 [37]. The correlation and principal component analysis were accomplished with Python using the scipy and scikit-learn packages. The matplotlib and seaborn Python packages were used for visualizations. The dataset is deposited at the NCBI Sequence Read Archive with accession number SRX3923124 [38].

### Quantitative Western blots

BSF cells were washed with cold PBS and lysed in reducing sample buffer (prepared as Invitrogen formulation but bromophenol Blue was replaced by 0.2% (W/V) Orange G, to reduce Licor background, and containing 0.1 M final DTT). Samples (equivalent to 1x10^6^ parasites) were loaded into a pre-cast Novex 4–12% Bis-Tris gel and separated using 1x MOPS running buffer (Invitrogen) for about 1 h at 200 V. Proteins were transferred onto nitrocellulose membrane using iBLOT system (Invitrogen), program 3 for 7 minutes. Membranes were placed in SNAPid cassette (Millipore) and blocked by filtration with 30 ml blocking buffer (50 mM Tris Base pH 7.4, 0.15M NaCl, 0.25% (w/v) BSA, 0.05% Tween 20, 0.05 NaN_3_ and 2% fish skin gelatine, pre-filtered at 0.2 μm). Primary antibodies (mouse anti-Cathepsin L and rabbit anti-HSP70) were diluted 1:1,000 in blocking buffer and rotated for 1h at RT inside SNAPid cassette. After incubation, membranes were washed three times with PBS containing 0.1% (w/v) Tween-20 (PBST) using SNAPid vacuum manifold. Anti-mouse green secondary antibody was diluted 1:15,000 while anti-rabbit red secondary was diluted 1:20,000 in blocking buffer and incubated with the membranes for 10 min at RT inside of SNAPid cassette. Membranes were washed in PBST and scanned using Licor Odyssey system.

## Supporting information

S1 Fig Creation of the *TbCMT1* conditional null mutant strategy. The strategy used to create the *TbCMT1* conditional null mutant from the Lister 427 BSF cell line is shown in (A). The first allele *TbCMT1* was replaced with a PAC resistance cassette. An ectopic copy of tetracycline-inducible *TbCMT1* with three C-terminal c-MYC tags was introduced into the rDNA locus. *TbCMT-MYC*_*3*_ was under the control of a procyclin promoter regulated by two tet-operator (TetO) sequences. The selection was via a phleomycin resistance marker driven by a T7 promoter. After induction of the ectopic copy of *TbCMT1-MYC*_*3*_ with tetracycline, the second *TbCMT1* allele was replaced with an HYG resistance cassette. (B) Southern blot analysis of the bloodstream form *TbCMT1* mutants. Genomic DNA (gDNA) was extracted from: lane 1, the parental SM (*TbCMT1*^+/+^) cells; lane 2, heterozygous cells with one *TbCMT1* allele replaced with *PAC* and an ectopic tetracycline inducible copy of *TbCMT1-MYC*_*3*_ introduced (*TbCMT1*^-/+^, *TbCMT1*^Ti^); and lane 3 the same cells following replacement of the second *TbCMT1* allele with *HYG* (*TbCMT1*^-/-^, *TbCMT1*^Ti^). Genomic DNA (gDNA) was digested with PstI and XhoI. Diagrams indicate predicted gDNA fragments for the native *TbCMT1* locus before and after replacement with antibiotic resistance genes. Southern blots were hybridised with a *TbCMT1* ORF probe (top panel), a *PAC* probe (middle panel), and a *HYG* probe (bottom panel). Note: SM cell line carries a fragment of the *HYG* gene which weakly hybridises to the HYG probe (lanes 1 and 2). When Wirtz and colleagues [17] generated the SM cell line, a fragment of the hygromycin gene used to select for the TetR construct remained in the genome. Consequently, an additional band is present at about 1.3 kb on the Southern blot hybridised to the HYG probe (bottom panel).
(PDF)

S2 Fig Southern blot analysis of *TbCMT1* null mutant. gDNA was extracted from parental SM (*TbCMT1*^+/+^) cells (lane 1), cells with one *TbCMT1* allele replaced with *PAC* (*TbCMT1*^-/+^) (lane 2), and cells following replacement of the second *TbCMT1* allele with *HYG* (*TbCMT1*^-/-^) (lane 3). gDNA was digested with PstI and XhoI. Diagrams indicate the predicted gDNA fragments for the native *TbCMT1* locus before and after replacement with antibiotic resistance genes. Southern blots were hybridised with *TbCMT1* ORF probe (top panel), *PAC* probe (middle panel), and *HYG* probe (bottom panel). When Wirtz and colleagues [17] generated the SM cell line, a fragment of the hygromycin gene used to select for the TetR construct remained in the genome. Consequently, an additional band is present at about 1.3 kb on the Southern blot hybridised to the HYG probe (bottom panel).
(PDF)

S3 Fig RNA-Seq biological replicate analyses. The correlations of the WT (A) and *TbCMT1* null (B) biological replicates were visualised by scatter plot matrices using the log10 value of the gene counts. Each matrix is composed of four rows and four columns to compare each biological replicate of wild type (WT1 to WT4) and *TbCMT1* null (null-1 to null-4) samples with each other. The diagonals of the matrices show the histograms of the log10 counts for each sample. The lower parts of the matrices diagonal show the kernel density plots of each paired sample, with the Pearson correlation coefficient reported in the top left corner of each paired plot. The upper parts of the matrices show the scatter plots for each paired sample.
(PDF)

S4 Fig Principal component analysis of the biological replicates. The gene expression variances between wild type (WT1 to WT4) and TbCMT1 null cells (null-1 to null-4) are displayed as a principal component analysis (PCA) of scaled log2-transformed transcript counts. The closer the points are in this two-dimensional space, the more similar the transcriptomes.
(PDF)

S5 Fig Quantitative Western blot for Cathepsin-L. Representative Western blot analysis of Cathepsin-L protein (red band) and HSP-70 protein (green band) in extracts of wild-type “single marker” (SM) cells and three independent *TbCMT1* null mutant clones (“double knockouts” null-1, null-2 and null-3). The positions of molecular weight markers are shown on the left.
(PDF)

S1 Table Differential gene expression analysis. The table reports the standard DESeq2 output of the gene expression analysis. The Gene identification number (Gene Id), the gene description (Gene Description), the DESeq2 baseMean counts (Base Mean), the log2 fold change (log2 Fold Change) and the adjusted p-values (Adjusted pValue) are reported. Note: The gene Tb10.v4.0040, located in a genome contig TP3F6-6f06.p1k, is identical in sequence to *TbCMT1* (Tb927.10.4500) and is presumably the same gene.
(XLSX)
