# Supplementary figures and images for: The mRNA cap methyltransferase gene TbCMT1 is not essential in vitro but is a virulence factor in vivo for bloodstream form Trypanosoma brucei
Source: PLoS One. 2018 Jul 24;13(7):e0201263. doi: 10.1371/journal.pone.0201263 (PMC6057678; doi:10.1371/journal.pone.0201263)

A

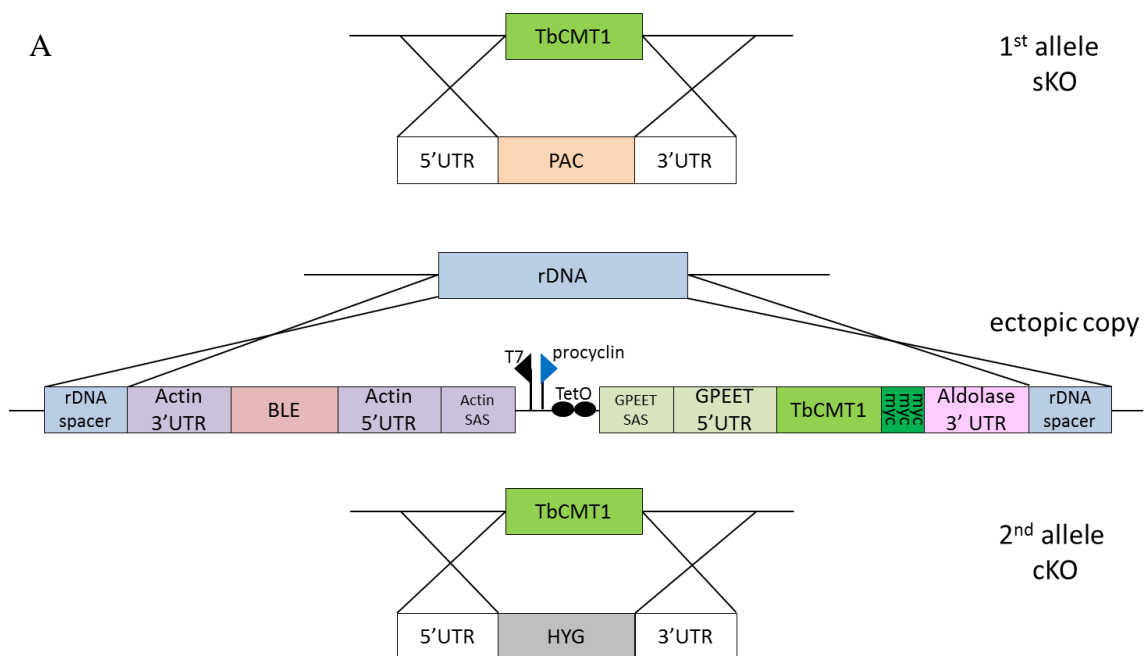

B

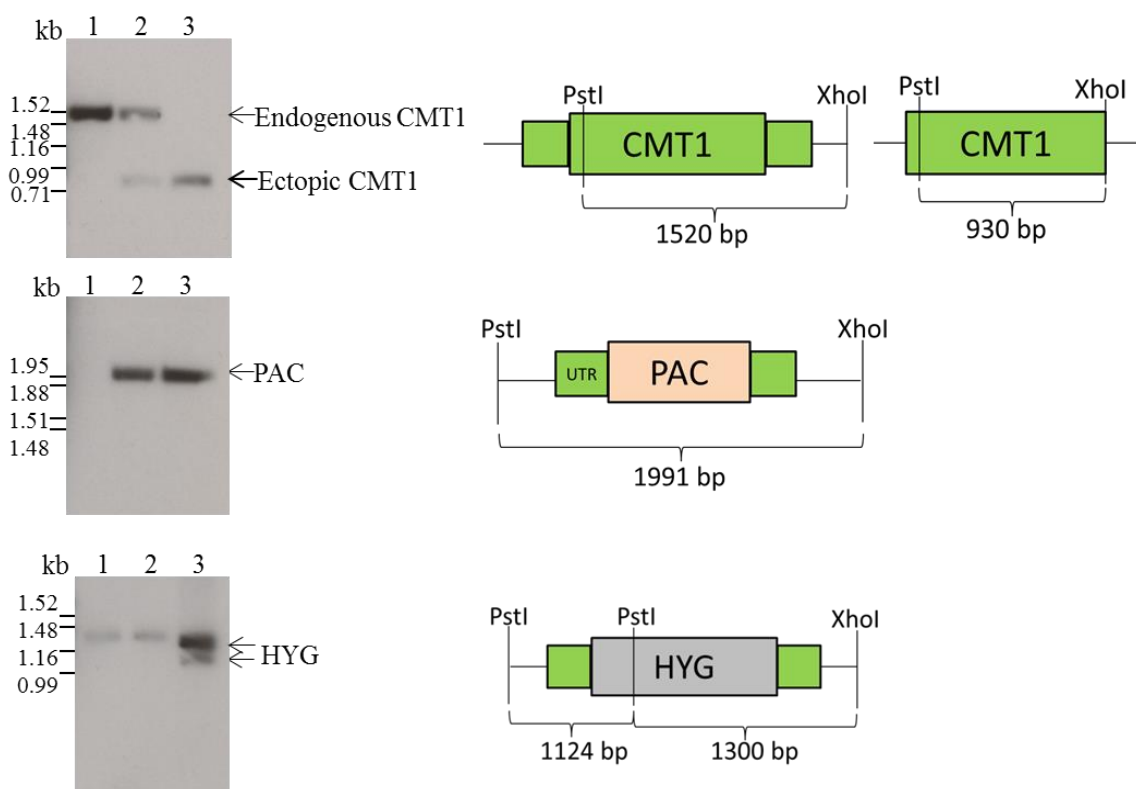

Supplement: S1 Fig — The strategy used to create the TbCMT1 conditional null mutant from the Lister 427 BSF cell line is shown in (A). The first allele TbCMT1 was replaced with a PAC resistance cassette. An ectopic copy of tetracycline-inducible TbCMT1 with three C-terminal c-MYC tags was introduced into the rDNA locus. TbCMT-MYC3 was under the control of a procyclin promoter regulated by two tet-operator (TetO) sequences. The selection was via a phleomycin resistance marker driven by a T7 promoter. After induction of the ectopic copy of TbCMT1-MYC3 with tetracycline, the second TbCMT1 allele was replaced with an HYG resistance cassette. (B) Southern blot analysis of the bloodstream form TbCMT1 mutants. Genomic DNA (gDNA) was extracted from: lane 1, the parental SM (TbCMT1+/+) cells; lane 2, heterozygous cells with one TbCMT1 allele replaced with PAC and an ectopic tetracycline inducible copy of TbCMT1-MYC3 introduced (TbCMT1-/+, TbCMT1Ti); and lane 3 the same cells following replacement of the second TbCMT1 allele with HYG (TbCMT1-/-, TbCMT1Ti). Genomic DNA (gDNA) was digested with PstI and XhoI. Diagrams indicate predicted gDNA fragments for the native TbCMT1 locus before and after replacement with antibiotic resistance genes. Southern blots were hybridised with a TbCMT1 ORF probe (top panel), a PAC probe (middle panel), and a HYG probe (bottom panel). Note: SM cell line carries a fragment of the HYG gene which weakly hybridises to the HYG probe (lanes 1 and 2). When Wirtz and colleagues [17] generated the SM cell line, a fragment of the hygromycin gene used to select for the TetR construct remained in the genome. Consequently, an additional band is present at about 1.3 kb on the Southern blot hybridised to the HYG probe (bottom panel). (PDF) [file pone.0201263.s001.pdf]

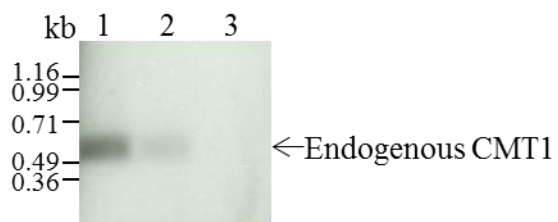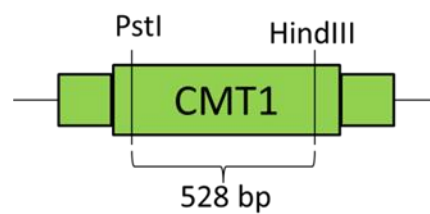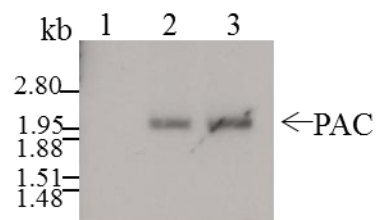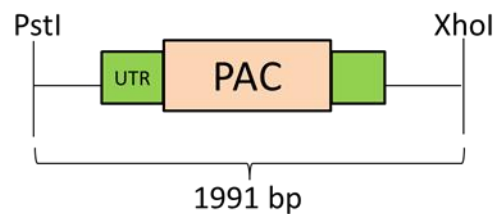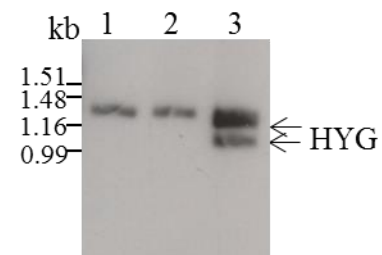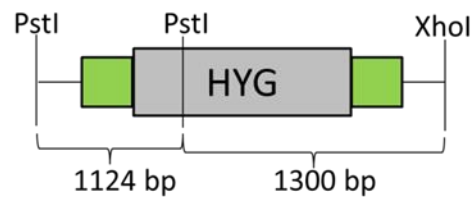

Supplement: S2 Fig — gDNA was extracted from parental SM (TbCMT1+/+) cells (lane 1), cells with one TbCMT1 allele replaced with PAC (TbCMT1-/+) (lane 2), and cells following replacement of the second TbCMT1 allele with HYG (TbCMT1-/-) (lane 3). gDNA was digested with PstI and XhoI. Diagrams indicate the predicted gDNA fragments for the native TbCMT1 locus before and after replacement with antibiotic resistance genes. Southern blots were hybridised with TbCMT1 ORF probe (top panel), PAC probe (middle panel), and HYG probe (bottom panel). When Wirtz and colleagues [17] generated the SM cell line, a fragment of the hygromycin gene used to select for the TetR construct remained in the genome. Consequently, an additional band is present at about 1.3 kb on the Southern blot hybridised to the HYG probe (bottom panel). (PDF) [file pone.0201263.s002.pdf]

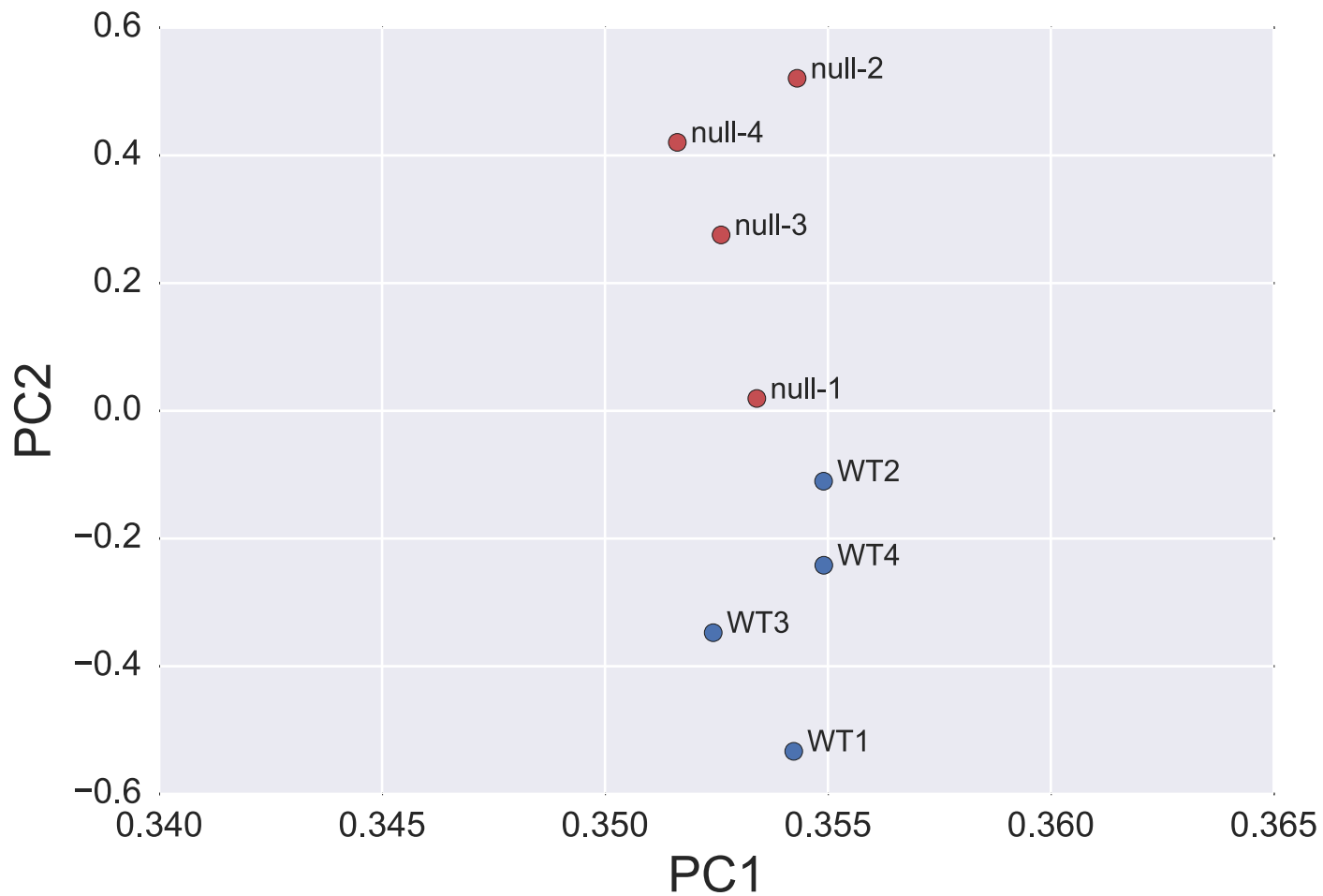

Supplement: S4 Fig — The gene expression variances between wild type (WT1 to WT4) and TbCMT1 null cells (null-1 to null-4) are displayed as a principal component analysis (PCA) of scaled log2-transformed transcript counts. The closer the points are in this two-dimensional space, the more similar the transcriptomes. (PDF) [file pone.0201263.s004.pdf]

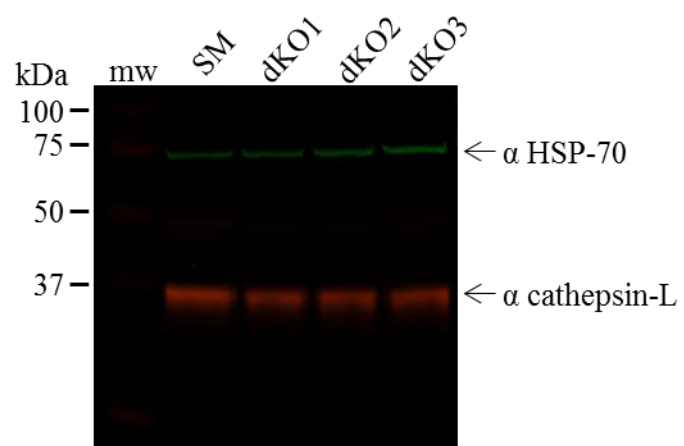

Supplement: S5 Fig — Representative Western blot analysis of Cathepsin-L protein (red band) and HSP-70 protein (green band) in extracts of wild-type “single marker” (SM) cells and three independent TbCMT1 null mutant clones (“double knockouts” null-1, null-2 and null-3). The positions of molecular weight markers are shown on the left. (PDF) [file pone.0201263.s005.pdf]
