# Supplementary material for: The mRNA cap methyltransferase gene TbCMT1 is not essential in vitro but is a virulence factor in vivo for bloodstream form Trypanosoma brucei
Source: PLoS One. 2018 Jul 24;13(7):e0201263. doi: 10.1371/journal.pone.0201263 (PMC6057678; doi:10.1371/journal.pone.0201263)
Supplement: S3 Fig — The correlations of the WT (A) and TbCMT1 null (B) biological replicates were visualised by scatter plot matrices using the log10 value of the gene counts. Each matrix is composed of four rows and four columns to compare each biological replicate of wild type (WT1 to WT4) and TbCMT1 null (null-1 to null-4) samples with each other. The diagonals of the matrices show the histograms of the log10 counts for each sample. The lower parts of the matrices diagonal show the kernel density plots of each paired sample, with the Pearson correlation coefficient reported in the top left corner of each paired plot. The upper parts of the matrices show the scatter plots for each paired sample. (PDF) [file pone.0201263.s003.pdf]

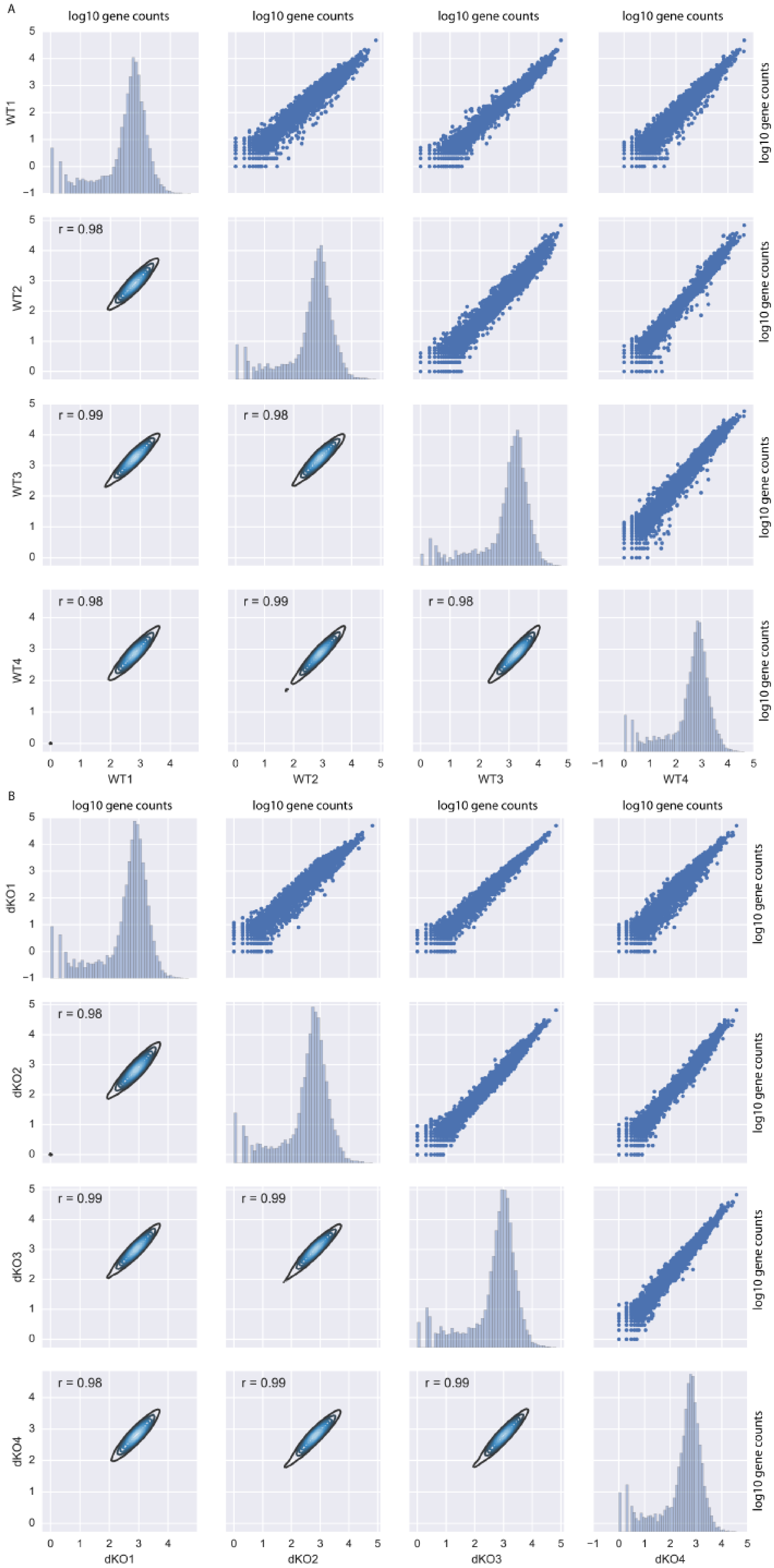
A
log10 gene counts
WT1
WT2
WT3
WT4
r = 0.98
r = 0.99
r = 0.98
r = 0.98
r = 0.99
r = 0.98
B
log10 gene counts
dKO1
dKO2
dKO3
dKO4
r = 0.98
r = 0.99
r = 0.99
r = 0.98
r = 0.99
r = 0.99
